# Novel prognostic nomograms for postoperative patients with oral cavity squamous cell carcinoma in the central region of China

**DOI:** 10.1186/s12885-024-12465-6

**Published:** 2024-06-14

**Authors:** Xue-Lian Xu, Jin-Hong Xu, Jia-Qi He, Yi-Hao Li, Hao Cheng

**Affiliations:** 1 Department of Radiotherapy Oncology, The First Affiliated Hospital of Xinxiang Medical University, 88 Jiankang Road, Xinxiang, 453100 Henan China; 2 Department of Otolaryngology, AnYang District Hospital, Anyang, 455000 Henan China; 3 Department of Radiotherapy Oncology, Affiliated Cancer Hospital of Zhengzhou University, Zhengzhou, 450000 Henan China

**Keywords:** Oral cavity squamous cell carcinoma, Dynamic nomogram, Postoperative, Prognosis, Risk stratification analysis

## Abstract

**Background:**

Oral cavity squamous cell carcinoma (OCSCC) is the most common pathological type in oral tumors. This study intends to construct a novel prognostic nomogram model based on China populations for these resectable OCSCC patients, and then validate these nomograms.

**Methods:**

A total of 607 postoperative patients with OCSCC diagnosed between June 2012 and June 2018 were obtained from two tertiary medical institutions in Xinxiang and Zhengzhou. Then, 70% of all the cases were randomly assigned to the training group and the rest to the validation group. The endpoint time was defined as overall survival (OS) and disease-free survival (DFS). The nomograms for predicting the 3-, and 5-year OS and DFS in postoperative OCSCC patients were established based on the independent prognostic factors, which were identified by the univariate analysis and multivariate analysis. A series of indexes were utilized to assess the performance and net benefit of these two newly constructed nomograms. Finally, the discrimination capability of OS and DFS was compared between the new risk stratification and the American Joint Committee on Cancer (AJCC) stage by Kaplan-Meier curves.

**Results:**

607 postoperative patients with OCSCC were selected and randomly assigned to the training cohort (*n* = 425) and validation cohort (*n* = 182). The nomograms for predicting OS and DFS in postoperative OCSCC patients had been established based on the independent prognostic factors. Moreover, dynamic nomograms were also established for more convenient clinical application. The C-index for predicting OS and DFS were 0.691, 0.674 in the training group, and 0.722, 0.680 in the validation group, respectively. Besides, the calibration curve displayed good consistency between the predicted survival probability and actual observations. Finally, the excellent performance of these two nomograms was verified by the NRI, IDI, and DCA curves in comparison to the AJCC stage system.

**Conclusion:**

The newly established and validated nomograms for predicting OS and DFS in postoperative patients with OCSCC perform well, which can be helpful for clinicians and contribute to clinical decision-making.

**Supplementary Information:**

The online version contains supplementary material available at 10.1186/s12885-024-12465-6.

## Introduction

Cancer of the oral cavity is the sixth most common neoplasm worldwide and has become a global health problem because of the comparatively high incidence and mortality [1, 2]. A series of tumors occurred in the anterior two-thirds of the tongue, the lips, hard and soft palate, gingivae, floor of the mouth, and oral mucosal were included in oral cancer [3]. Oral cavity squamous cell carcinoma (OCSCC) is the most common histological type and accounts for 90% of oral cancers [4]. Generally, the incidence of OCSCC is high in low- and middle-income countries [5, 6], especially in Asia countries and male populations due to the prevalence of risk habits, such as tobacco smoking, tobacco chewing, alcohol intake, betel quid use, oral microbiome, and poor nutritional status [7–12]. The preferred treatment for oral cancer is surgical treatment. Although improved surgical techniques combined with postoperative radiation or chemo-radiation therapy have been routinely applied, the 5-year survival rate has still been at a low level for the last few decades due to the high local recurrence rate and cervical lymph node metastasis rate [13–16]. Usually, the prognosis prediction was mainly according to the American Joint Committee on Cancer (AJCC) TNM staging system in both resectable and inoperable patients. There was a dramatic difference for those patients in the prognosis and treatment strategy in practice. The deficiency of the traditional AJCC stage system was common in clinical applications. Therefore, it is imperative to identify the predictors specifically for resectable OCSCC patients and establish an efficient prognosis prediction model to accurately predict the prognosis, and then benefit clinicians and patients.

A nomogram is a frequently-used and convenient tool in the prognosis prediction of various cancers [17–19]. Currently, the AJCC stage system is the most important prognosis prediction tool, which provides an estimate of prognosis based on the anatomic range. However, there are still various clinicopathological factors that have not been incorporated and taken into full consideration [20, 21], such as age [22], quit smoking or not after surgical resection [23], postoperative resection margin [24], eastern cooperative oncology group performance status (ECOG PS) score [25], immune/inflammation index [26], comorbidity [27], and complication [28], etc. Tagliabue Marta et al. [29] revealed that younger oral cancer patients had a higher risk of local recurrence but a better overall survival compared with older patients. A meta-analysis study indicates that platelet-to-lymphocyte ratio (PLR) is a poor progression factor in oral cancer [30]. Previous studies have found that immune-inflammation-related indexes play an important role in prognosis and the efficacy of conventional therapies for oral cancer [31, 32], such as the systemic immune-inflammation index (SII), prognostic nutrition index (PNI), neutrophil-to-lymphocyte ratio (NLR), and Glasgow prognostic score (GPS). Besides, comorbidity has an important influence on the prognosis of head and neck tumor [33, 34]. The age-adjusted Charlson comorbidity index (ACCI) is a measure of comorbidity and an important predictor in various tumors [35, 36]. The nomogram model can take the above clinicopathological factors into account in comparison with the AJCC stage system, and predict the prognosis individually for each patient.

In this study, nomograms for predicting overall survival (OS) and disease-free survival (DFS) were developed and validated in postoperative patients with OCSCC based on two medical centers, which can be helpful for personalized prognosis prediction and clinical management.

## Materials and methods

### Materials and clinicopathological factors

**Fig. 1:**
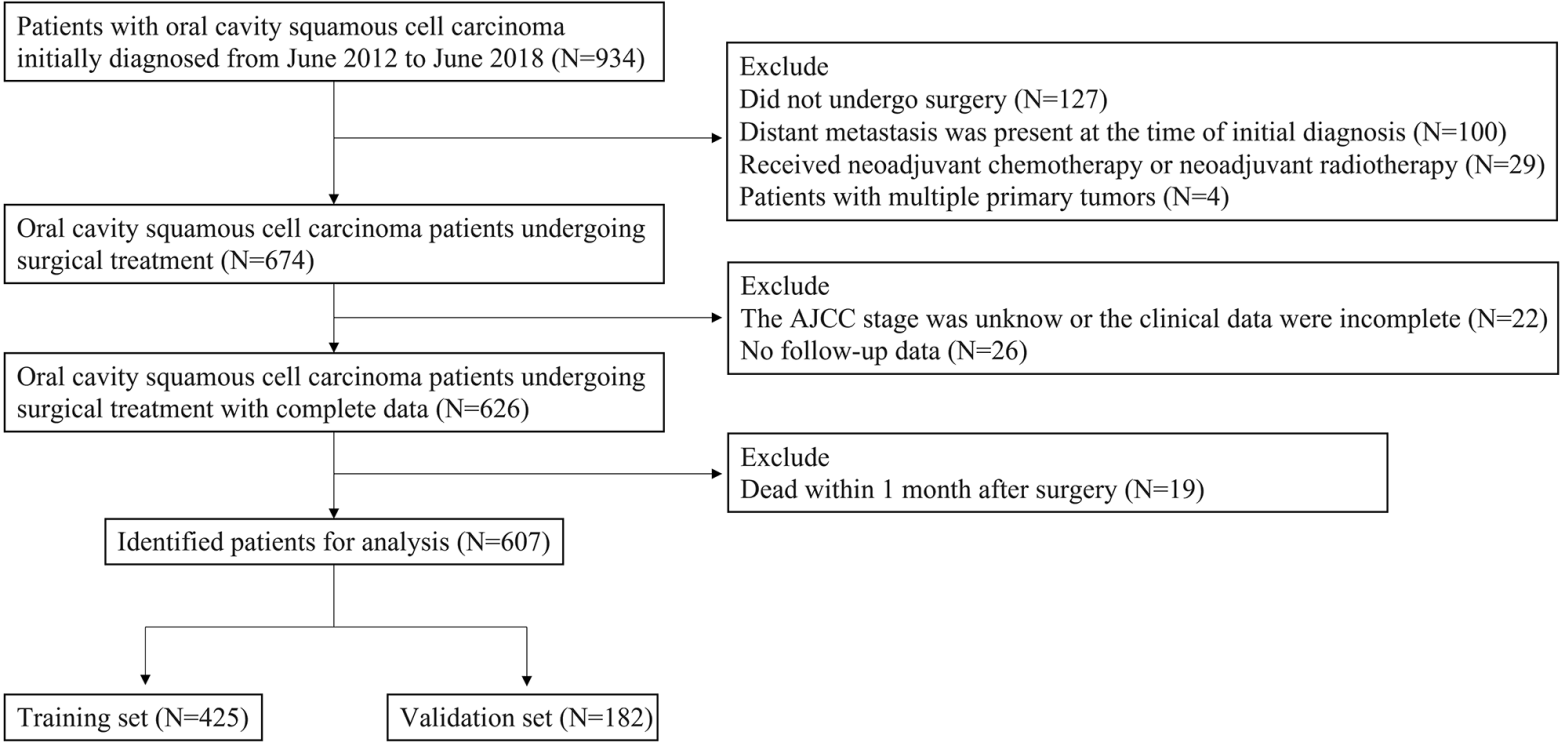
Flow diagram of the postoperative oral cavity squamous cell carcinoma (OCSCC) patients based on the inclusion and exclusion criteria

A total of 607 postoperative OCSCC patients diagnosed between June 2012 and June 2018 were included in this study from two medical institutions, including (1) the First Affiliated Hospital of Xinxiang Medical University and (2) the Affiliated Cancer Hospital of Zhengzhou University. Cases were obtained according to the inclusion and exclusion criteria as follows. The inclusion criteria include (1) malignant Oral cavity squamous cell carcinoma (OCSCC) cases and pathologically confirmed; (2) Age at diagnosis ≥ 18; (3) single primary malignant tumor; (4) active follow-up. The exclusion criteria include: (1) No surgical treatment was performed; (2) There was distant metastasis at first diagnosis; (3) Received neoadjuvant chemotherapy or neoadjuvant radiotherapy; (4) Patients with multiple primary tumors; (5) TNM stage was unknown or clinical data was incomplete; (6) Death occurred within 1 month after surgery. The flow chart is displayed in Fig. 1.

All cases were randomly divided at a ratio of 7:3 into training and validation groups by SPSS 20.0. Nomograms were conducted based on the training cohort, and the validation of nomograms was based on the validation cohort. There are 20 clinicopathological variables of postoperative OCSCC patients were incorporated into the analysis, including age at diagnosis, gender, grade, AJCC stage, surgical margin, vascular invasion (VI), perineural invasion, smoking after surgery, extranodal extension (ENE), body mass index (BMI), hemoglobin (HGB), ECOG PS core; GPS; SII; PNI; PLR; NLR; ACCI; adjuvant radiotherapy; adjuvant chemotherapy.

The radiotherapy dose ranged from 50 to 72.5 Gy. Paclitaxel, platinum, and fluorouracil were the main drugs applied for adjuvant chemotherapy. The ACCI is a comprehensive evaluation of various comorbidities and is calculated by the sum of the comorbidity score and the age score. Table S1 & Table S2 showed the calculation method for ACCI, GPS, PNI, NLR, PLR, and BMI. The cutoff points for SII, PNI, PLR, and NLR were decided by the X-tile software. The OS and DFS were considered as endpoints.

### Statistical analysis

R software (version 4.2.2) and SPSS 20.0 were utilized in this study for statistical analyses in this study. The Chi-square test and independent-sample T-test were utilized to compare the differences in the baseline characteristics between the training and validation groups. The associations between clinicopathological variables and prognosis were determined by Univariate Cox regression analysis. Then, the multivariate Cox regression analysis was utilized to identify the independent predictors for postoperative OCSCC patients (*P* < 0.05). Finally, these identified independent prognostic factors were applied to establish nomograms for predicting OS and DFS in postoperative OCSCC patients.

R software was utilized to calculate the calibration curve, the concordance index (C-index), the receiver operating characteristic (ROC), the decision curve (DCA), the integrated discrimination improvement (IDI), and the net reclassification improvement (NRI). The discriminative capability and the prediction efficiency of the model were evaluated by C-index, ROC, and the calibration curve. Besides, DCA, IDI, and NRI were brought to appraise the predictive power and potency of the nomogram compared to the AJCC stage system. Finally, new risk stratification was established based on the total points of the nomogram, and all patients were divided into low, medium, and high-risk groups by X-tile software. Log-rank test and Kaplan-Meier plots were applied to compare the survival time in different risk stratification groups.

## Results

### Patient characteristics

A total of 607 postoperative patients with OCSCC were obtained and randomly divided into the training group (*n* = 425) and validation group (*n* = 182) at a ratio of 7:3 (Fig. 1). The median follow-up time was 34 months (1-130 months) for DFS, and 37 months (1-130 months) for OS. Table 1 displays the detailed clinicopathological factors of all selected cases. There was no significant difference in clinicopathological features between the training set and the validation set. The median age was 51 years old (range from 22 to 90). There was no significant difference in OCSCC morbidity between men and women (58.8% vs. 41.2%). OCSCC patients with AJCC Stage II (24.4%) & III (39.4%), negative surgical margin (89.6%), without vascular invasion (91.1%), without perineural invasion (89.1%), negative extranodal extension (93.7%), ECOG PS score between 0 and 1 (78.1%), Glasgow prognostic score equal to 0 (71.8%) were more likely to accept surgical treatment in clinical practice. OCSCC patients after surgical resection often give up smoking (87.5%). Most postoperative OCSCC patients underwent adjuvant radiotherapy (65.4%) and/or adjuvant chemotherapy (63.6%).

**Table 1:** Clinical information of postoperative OCSCC patients in the training and validation groups

Characteristics	All Patients (*n* = 607) *N* (%)	Training cohort (*n* = 425) *N* (%)	Validation cohort (*n* = 182) *N* (%)	*P*
**Age at diagnosis (years)**				0.199
Median (range)	51.0 (22–90)	51.0 (22–90)	50.0 (22–89)	
**Gender**				0.653
Male	357 (58.8%)	247 (58.1%)	110 (60.4%)	
Female	250 (41.2%)	178 (41.9%)	72 (39.6%)	
**Grade**				0.137
I	186 (30.6%)	136 (32.0%)	50 (27.5%)	
II	204 (33.6%)	143 (33.6%)	61 (33.5%)	
III	217 (35.7%)	146 (34.4%)	71 (39.0%)	
**AJCC Stage**				
I	107 (17.6%)	72 (16.9%)	35 (14.8%)	0.162
II	148 (24.4%)	100 (23.5%)	48 (20.3%)	
III	239 (39.4%)	164 (38.6%)	75 (42.9%)	
IVA & IVB	113 (18.6%)	89 (36.4%)	24 (22.0%)	
**Surgical margin**				0.562
Positive	63 (10.4%)	42 (9.9%)	21 (11.5%)	
Negative	544 (89.6%)	383 (90.1%)	161 (88.5%)	
**VI**				0.437
Yes	54 (8.9%)	35 (8.2%)	11 (10.4%)	
No	553 (91.1%)	390 (91.8%)	171 (89.6%)	
**Perineural invasion**				0.046
Yes	66 (10.9%)	39 (9.2%)	27 (14.8%)	
No	541 (89.1%)	386 (90.8%)	155 (85.2%)	
**Smoking after surgery**				0.593
Yes	76 (12.5%)	51 (12.0%)	25 (13.7%)	
No	531 (87.5%)	374 (88.0%)	157 (86.3%)	
**ENE**				0.585
Positive	38 (6.3%)	25 (5.9%)	13 (7.1%)	
Negative	569 (93.7%)	400 (94.1%)	169 (92.9%)	
**BMI (kg/m** ^**2**^ **)**				0.437
Median (range)	21.3 (16.0-32.9)	21.3 (16.0-32.9)	21.4 (16.6–31.8)	
**HGB (g/L)**				0.097
Median (range)	105.6 (71–164)	104.4 (71–164)	107.6 (71–162)	
**ECOG PS score**				0.454
0–1	474 (78.1%)	328 (77.2%)	146 (80.2%)	
2	133 (21.9%)	97 (22.8%)	36 (19.8%)	
**GPS**				0.081
0	436 (71.8%)	305 (71.8%)	131 (72.0%)	
1	115 (18.9%)	87 (20.5%)	28 (15.4%)	
2	56 (9.2%)	33 (7.8%)	23 (12.6%)	
**SII**				0.357
Median (IQR)	1167 (651–1582)	1166 (653–1586)	1194 (596–1556)	
**PNI**				0.424
Median (IQR)	70 (52–95)	69 (51–93)	73 (53–97)	
**PLR**				0.338
Median (IQR)	148 (93–210)	148 (94–217)	148 (90–200)	
**NLR**				0.766
Median (IQR)	2.42 (1.39–3.34)	2.45 (1.35–3.34)	2.41 (1.45–3.37)	
**ACCI**				0.743
2–3	252 (41.5%)	179 (42.1%)	73 (40.1%)	
4–5	204 (33.6%)	144 (33.9%)	60 (43.0%)	
≥ 6	151 (24.9%)	102 (24.0%)	49 (26.0%)	
**Adjuvant radiotherapy**				0.265
Yes	397 (65.4%)	284 (66.8%)	113 (62.1%)	
No	210 (34.6%)	141 (33.2%)	69 (37.9%)	
**Adjuvant chemotherapy**				0.520
Yes	386 (63.6%)	274 (64.5%)	112 (61.5%)	
No	221 (36.4%)	151 (35.5%)	70 (38.5%)	
**DFS (months)**				0.120
Median (range)	34 (1-130)	34 (1-129)	34 (1-130)	
**OS (months)**				0.177
Median (range)	37 (1-130)	36 (1-129)	39 (1-130)	

*Abbreviations* ACCI, age-adjusted Charlson comorbidity index; AJCC, American Joint Committee on Cancer; BMI, body mass index; DFS, disease-free survival; ECOG, eastern cooperative oncology group; ENE, extranodal extension; GPS, Glasgow prognostic Score; HGB, hemoglobin; IQR, interquartile range; LNM, lymph node metastasis; NLR, neutrophil-to-lymphocyte ratio; OS, overall survival; OCSCC; oral cavity squamous cell carcinoma; PLR, platelet-to-lymphocyte ratio; PNI, prognostic nutrition index; PS, performance status; RT, radiotherapy; SD, standard deviation; SII, systemic immune-inflammation index; VI, vascular invasion

### Independent prognostic variables in postoperative patients with OCSCC

A total of 15 clinicopathological factors were associated with OS of postoperative OCSCC patients by the univariate Cox regression analysis, including age at diagnosis, grade, AJCC stage, surgical margin, VI, perineural invasion, smoking after surgery, ENE, ECOG PS score, GPS, SII, PLR, ACCI, adjuvant radiotherapy and chemotherapy (*P* < 0.05). Additionally, there are 13 variables associated with DFS of postoperative patients with OCSCC, such as age at diagnosis, AJCC stage, surgical margin, VI, perineural invasion, smoking after surgery, ECOG PS score, GPS, SII, PLR, ACCI, adjuvant radiotherapy and chemotherapy (*P* < 0.05). Then, multivariate Cox analysis was applied to determine the independent predictors for OS and DFS, 9 variables of AJCC stage, perineural invasion, smoking after surgery, ECOG PS score, SII, PLR, ACCI, adjuvant radiotherapy and chemotherapy were determined as independent predictors for OS and DFS (*P* < 0.05). Finally, the nomograms for predicting the OS and DFS of postoperative OCSCC patients were developed based on these independent predictors, and Tables 2 and 3 displayed the details.

**Table 2:** Univariate and multivariate analyses of clinicopathologic parameters in postoperative patients with OCSCC for predicting OS

Characteristics	Univariate analysis		Multivariate analysis	
	HR (95% CI)	*P*	HR (95% CI)	*P*
**Age at diagnosis** (years)	1.009 (1.001–1.017)	**0.033**	1.000 (0.988–1.012)	0.937
**Gender**				
Female	Reference			
Male	0.837 (0.636–1.103)	0.206		
**Grade**				
I	Reference		Reference	
II	1.155 (0.824–1.621)	0.532	1.007 (0.711–1.426)	0.968
III	1.599 (1.143–2.238)	**0.025**	1.389 (0.977–1.977)	0.067
**AJCC stage**				
I	Reference		Reference	
II	1.185 (0.753–1.865)	0.462	1.211 (0.764–1.920)	0.415
III	1.822 (1.222–2.716)	**0.003**	1.907 (1.269–2.866)	**0.002**
IVA & IVB	1.935 (1.251–2.995)	**0.003**	2.156 (1.368–3.398)	**0.001**
**Surgical margin**				
Negative	Reference		Reference	
Positive	1.706 (1.143–2.548)	**0.009**	1.205 (0.685–2.120)	0.518
**VI**				
No	Reference		Reference	
Yes	1.769 (1.135–2.757)	**0.012**	0.985 (0.525–1.850)	0.963
**Perineural invasion**				
No	Reference		Reference	
Yes	1.852 (1.235–2.776)	**0.003**	2.271 (1.478–3.490)	**< 0.001**
**Smoking after surgery**				
No	Reference		Reference	
Yes	1.897 (1.284–2.803)	**0.001**	1.912 (1.282–2.852)	**0.001**
**ENE**				
Negative	Reference		Reference	
Positive	1.763 (1.057–2.942)	**0.030**	1.080 (0.605–1.927)	0.796
**BMI (kg/m** ^**2**^ **)**	1.020 (0.985–1.055)	0.262		
**HGB (g/L)**	0.999 (0.993–1.005)	0.745		
**ECOG PS score**				
0–1	Reference		Reference	
2	1.649 (1.213–2.241)	**0.001**	1.490 (1.085–2.046)	**0.014**
**GPS**				
0	Reference			
1	1.319 (0.942–1.847)	0.129		
2	2.219 (1.335–3.686)	0.089		
**SII**				
< 1433	Reference		Reference	
≥ 1433	1.618 (1.207–2.169)	**0.001**	1.551 (1.153–2.087)	**0.004**
**PNI**				
< 87	Reference			
≥ 88	0.768 (0.569–1.037)	0.085		
**PLR**				
< 185	Reference		Reference	
≥ 185	1.568 (1.165–2.111)	**0.003**	1.513 (1.108–2.066)	**0.009**
**NLR**				
< 2.95	Reference			
≥ 2.96	1.327 (0.942–1.847)	0.061		
**ACCI**				
2–3	Reference		Reference	
4–5	1.126 (0.816–1.553)	0.470	1.220 (0.874–1.703)	0.223
≥ 6	1.982 (1.400–2.805)	**< 0.001**	2.311 (1.602–3.332)	**< 0.001**
**Adjuvant radiotherapy**				
Yes	Reference		Reference	
No	0.676 (0.501–0.913)	**0.011**	0.669 (0.491–0.911)	**0.011**
**Adjuvant chemotherapy**				
Yes	Reference		Reference	
No	0.676 (0.502–0.910)	**0.010**	0.688 (0.505–0.937)	**0.018**

*Abbreviations* ACCI, age-adjusted Charlson comorbidity index; AJCC, American Joint Committee on Cancer; BMI, body mass index; CI, confidence interval; ECOG, eastern cooperative oncology group; ENE, extranodal extension; GPS, Glasgow prognostic score; HGB, hemoglobin; HR, hazard ratio; IQR, interquartile range; NLR, neutrophil-to-lymphocyte ratio; OS, overall survival; OCSCC; oral cavity squamous cell carcinoma; PLR, platelet-to-lymphocyte ratio; PNI, prognostic nutrition index; PS, performance status; SII, systemic immune-inflammation index; VI, vascular invasion

**Table 3:** Univariate and multivariate analyses of characteristics for predicting DFS in postoperative patients with OCSCC

Characteristics	Univariate analysis	*P*	Multivariate analysis	*P*
	HR (95% CI)		HR (95% CI)	
**Age at diagnosis** (years)	1.010 (1.003–1.018)	**0.007**	1.003 (0.636–1.970)	0.661
**Gender**				
Female	Reference			
Male	0.891 (0.686–1.156)	0.385		
**Grade**		0.068		
I	Reference		Reference	
II	1.159 (0.830–1.561)	0.420	1.061 (0.763–1.476)	0.725
III	1.392 (1.013–1.912)	**0.041**	1.392 (0.937–1.840)	0.113
**AJCC stage**				
I	Reference		Reference	
II	1.105 (0.726–1.681)	0.642	1.091 (0.714–1.669)	0.686
III	1.597 (1.098–2.323)	**0.014**	1.590 (1.087–2.326)	**0.017**
IVA & IVB	1.713 (1.135–2.584)	**0.010**	1.815 (1.187–2.777)	**0.006**
**Surgical margin**				
Negative	Reference		Reference	
Positive	1.785 (1.220–2.612)	**0.003**	1.308 (0.778–2.199)	0.312
**VI**				
No	Reference		Reference	
Yes	2.008 (1.333–3.024)	**0.001**	1.120 (0.636–1.970)	0.695
**Perineural invasion**				
No	Reference		Reference	
Yes	1.948 (1.320–2.876)	**0.001**	2.361 (1.567–3.558)	**< 0.001**
**Smoking after surgery**				
No	Reference		Reference	
Yes	1.792 (1.231–2.609)	**0.002**	1.748 (1.189–2.570)	**0.005**
**ENE**				
Negative	Reference			
Positive	1.541 (0.939–2.528)	0.087		
**BMI (kg/m** ^**2**^ **)**	1.017 (0.984–1.050)	0.312		
**HGB (g/L)**	1.004 (0.998–1.010)	0.171		
**ECOG PS score**				
0–1	Reference		Reference	
2	1.627 (1.218–2.172)	**0.001**	1.520 (1.130–2.044)	**0.006**
**GPS**				
0	Reference			
1	1.336 (0.974–1.831)	0.072		
2	1.349 (0.820–2.202)	0.231		
**SII**				
< 1433	Reference		Reference	
≥ 1433	1.499 (1.140–1.969)	**0.004**	1.457 (1.104–1.922)	**0.008**
**PNI**				
< 87	Reference			
≥ 88	0.776 (0.584–1.030)	0.079		
**PLR**				
< 185	Reference		Reference	
≥ 185	1.507 (1.142–1.989)	**0.004**	1.461 (1.092–1.954)	**0.011**
**NLR**				
< 2.95	Reference			
≥ 2.96	1.249 (0.948–1.645)	0.115		
**ACCI**				
2–3	Reference		Reference	
4–5	1.126 (0.816–1.553)	0.135	1.345 (0.987–1.833)	**0.061**
≥ 6	1.982 (1.400–2.805)	**< 0.001**	2.128 (1.512–2.995)	**< 0.001**
**Adjuvant radiotherapy**				
Yes	Reference		Reference	
No	0.717 (0.542–0.950)	**0.020**	0.720 (0.538–0.963)	**0.027**
**Adjuvant chemotherapy**				
Yes	Reference		Reference	
No	0.703 (0.532–0.927)	**0.013**	0.729 (0.546–0.973)	**0.032**

*Abbreviations* ACCI, age-adjusted Charlson comorbidity index; AJCC, American Joint Committee on Cancer; BMI, body mass index; CI, confidence interval; DFS, disease-free survival; ECOG, eastern cooperative oncology group; ENE, extranodal extension; GPS, Glasgow prognostic Score; HGB, hemoglobin; HR, hazard ratio; IQR, interquartile range; NLR, neutrophil-to-lymphocyte ratio; OCSCC; oral cavity squamous cell carcinoma; PLR, platelet-to-lymphocyte ratio; PNI, prognostic nutrition index; PS, performance status; SII, systemic immune-inflammation index; VI, vascular invasion

### Construction and validation of the nomograms

**Fig. 2:**
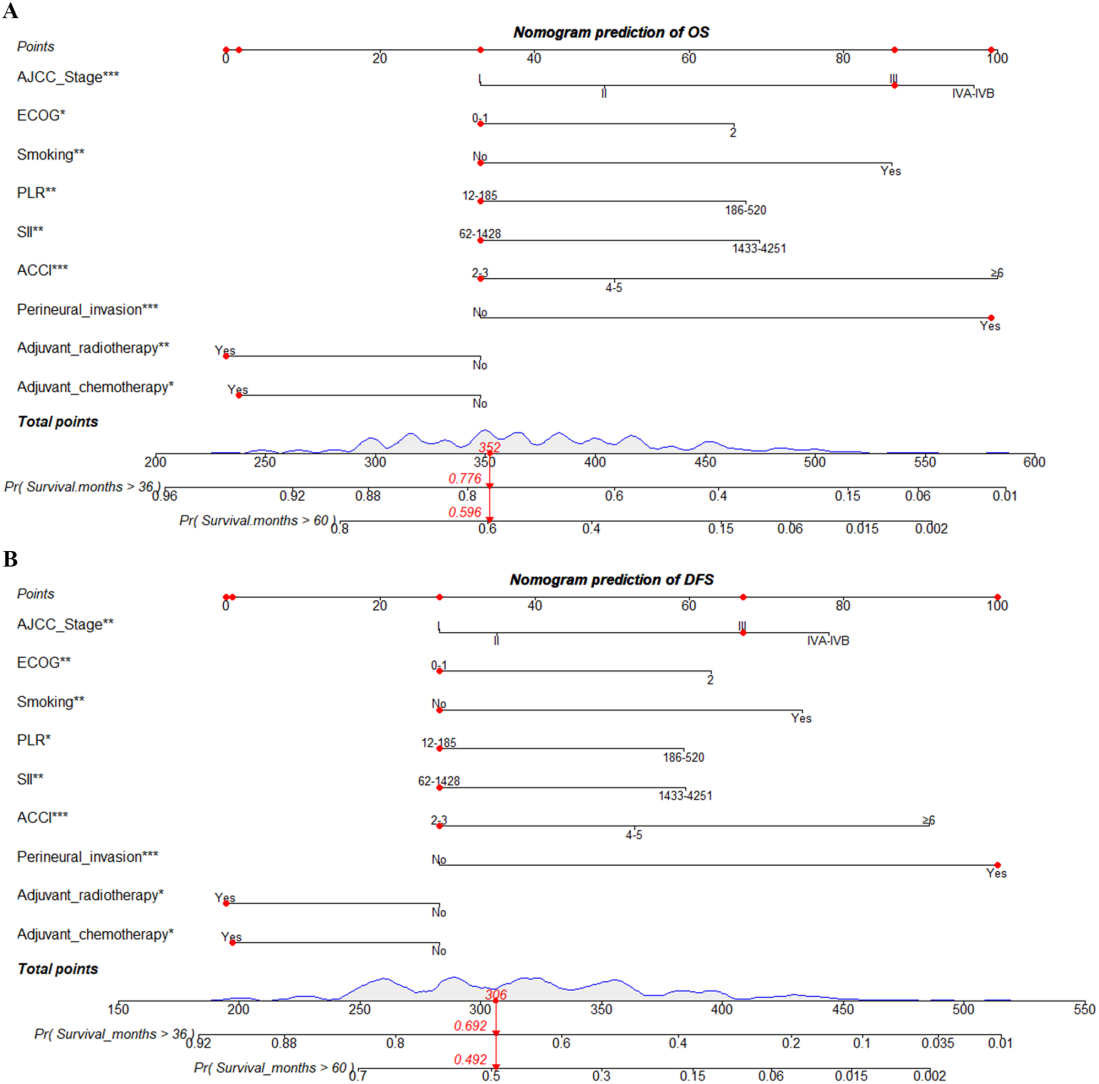
Nomograms to predict 3-, and 5-year overall survival (**A**) and disease-free survival (**B**) for postoperative OCSCC patients. *P < 0.05, **P < 0.01, ***P < 0.001. *Abbreviations* ACCI, age-adjusted Charlson comorbidity index; AJCC, American Joint Committee on Cancer; ECOG, eastern cooperative oncology group; OCSCC, oral cavity squamous cell carcinoma; PLR, platelet-to-lymphocyte ratio; SII, systemic immune-inflammation index

The nomograms were built based on the identified independent clinical factors for predicting the 3-, and 5-year OS and DFS in postoperative OCSCC patients. According to the nomograms constructed, the 3-, and 5-year probability of OS and DFS could be acquired by calculating the total score of all independent variables. The OS and DFS probability for the given example patient were displayed in Fig. 2A **&** Fig. 2B. Additionally, the dynamic web-based calculator was also developed according to the model, in order to simplify the application of these two nomograms (web-based calculator for OS: https://xxlchxjh.shinyapps.io/DynNomapp_oral_cancer_OS/; web-based calculator for DFS: https://xxlchxjh.shinyapps.io/DynNomapp_oral_cancer_DFS/). The online calculator can calculate the survival probability expediently by clinicians.

**Fig. 3:**
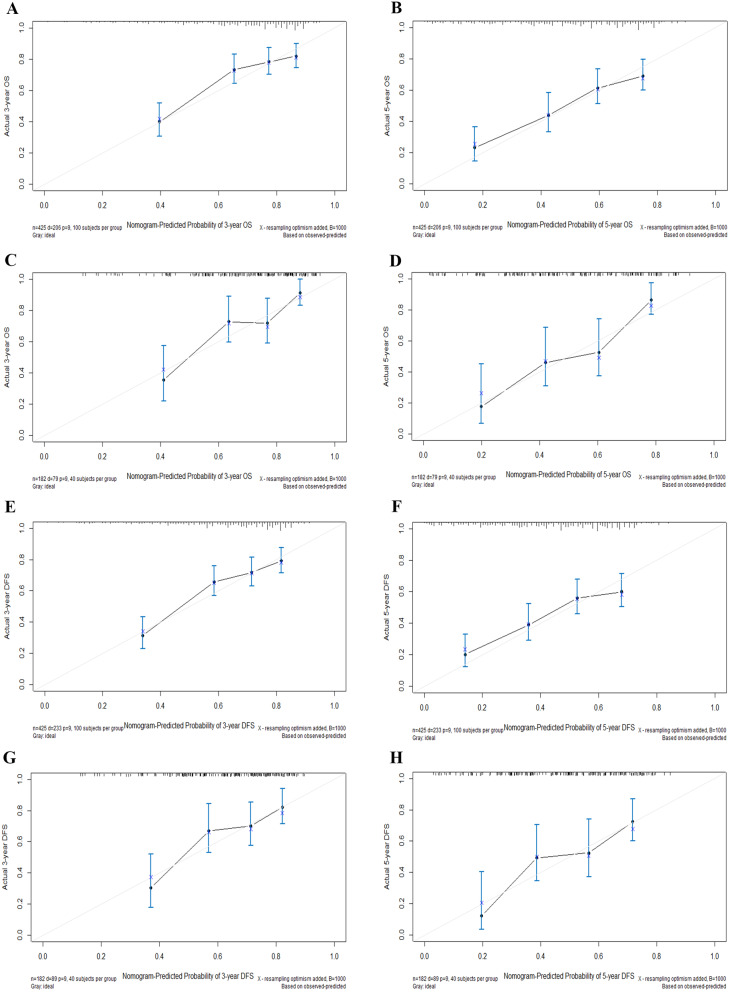
Calibration plots of 3-, and 5-year OS (**A**-**D**) and DFS (**E**-**H**) for postoperative OCSCC patients. (**A**, **B**) Calibration plots of 3-, and 5-year OS in the training cohort. (**C**, **D**) Calibration plots of 3-, and 5-year OS in the validation cohort. (**E**, **F**) Calibration plots of 3-, and 5-year DFS in the training cohort. (**G**, **H**) Calibration plots of 3-, and 5-year DFS in the validation cohort. *Abbreviations* DCA, decision curve analysis; DFS, disease-free survival; OS, overall survival; OCSCC, oral cavity squamous cell carcinoma

The performance of the developed nomograms was evaluated by the C-index and calibration curve. The C-index for the prediction of OS was 0.691 (95% CI: 0.650–0.732) in the training cohort and 0.722 (95% CI: 0.661–0.783) in the validation cohort, respectively. Besides, the C-index was 0.674 (95% CI: 0.635–0.713) and 0.680 (95% CI: 0.617–0.743) for predicting DFS in the training and validation groups (Table 4). Additionally, there was also a good consistency shown by the calibration curves for the training and validation sets (Fig. 3).

**Table 4:** The NRI, IDI, and C-index of the nomograms and AJCC Stage system in OS and DFS prediction for postoperative patients with OCSCC

Index	Training cohort			Validation cohort		
	Estimate	95%CI	*P*	Estimate	95%CI	*P*
**NRI (vs. AJCC** **Stage system)**						
For 3-year OS	0.287	0.224–0.432		0.410	0.154–0.586	
For 5-year OS	0.239	0.193–0.425		0.434	0.207–0.604	
For 3-year DFS	0.284	0.211–0.428		0.332	0.162–0.507	
For 5-year DFS	0.196	0.132–0.372		0.427	0.166–0.564	
**IDI (vs. AJCC** **Stage system)**						
For 3-year OS	0.151	0.115–0.241	< 0.001	0.137	0.063–0.269	< 0.001
For 5-year OS	0.151	0.103–0.221	< 0.001	0.170	0.076–0.289	< 0.001
For 3-year DFS	0.147	0.106–0.220	< 0.001	0.123	0.047–0.228	< 0.001
For 5-year DFS	0.121	0.081–0.193	< 0.001	0.123	0.054–0.236	< 0.001
**C-index**						
The nomogram (OS)	0.691	0.650–0.732		0.722	0.661–0.783	
The nomogram (DFS)	0.674	0.635–0.713		0.680	0.617–0.743	
The AJCC Stage (OS)	0.580	0.539–0.621		0.554	0.487–0.621	
The AJCC Stage (DFS)	0.591	0.548–0.634		0.553	0.482–0.623	

*Abbreviations* AJCC, American joint committee on cancer; CI, confidence interval; C-index, concordance index; DFS, disease-free survival; IDI, integrated discrimination improvement; NRI, net reclassification index; OS, overall survival; OCSCC, oral cavity squamous cell carcinoma

**Fig. 4:**
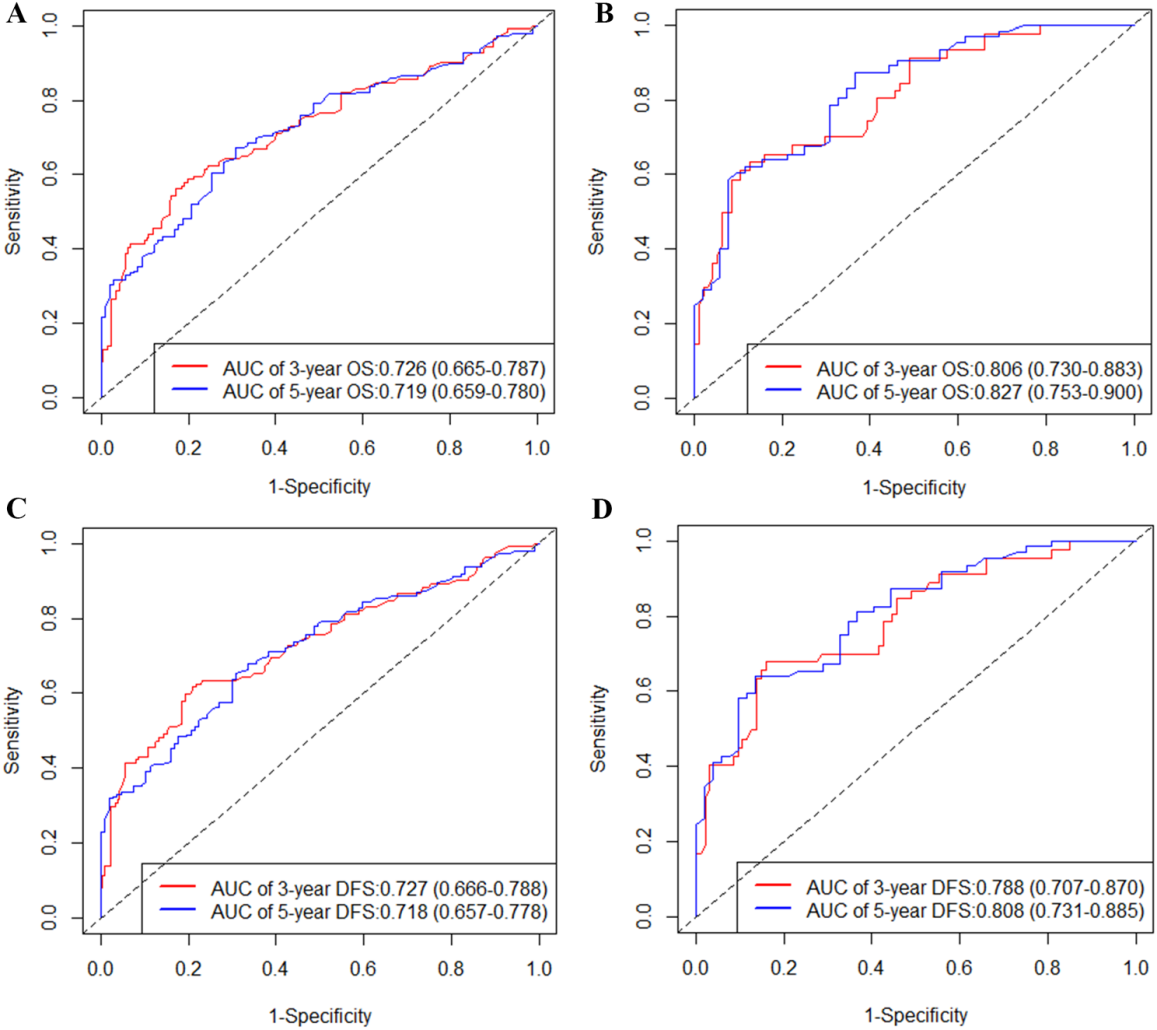
Time-dependent ROC curves of the nomogram for 3-, and 5-year predictions. AUC for predicting OS in the training set (**A**) and validation set (**B**); ROC curves corresponding to DFS in the training (**C**) and validation cohort (**D**), respectively. *Abbreviations* AUC, area under curve; DFS, disease-free survival; OS, overall survival; ROC, receiver operating characteristic

**Fig. 5:**
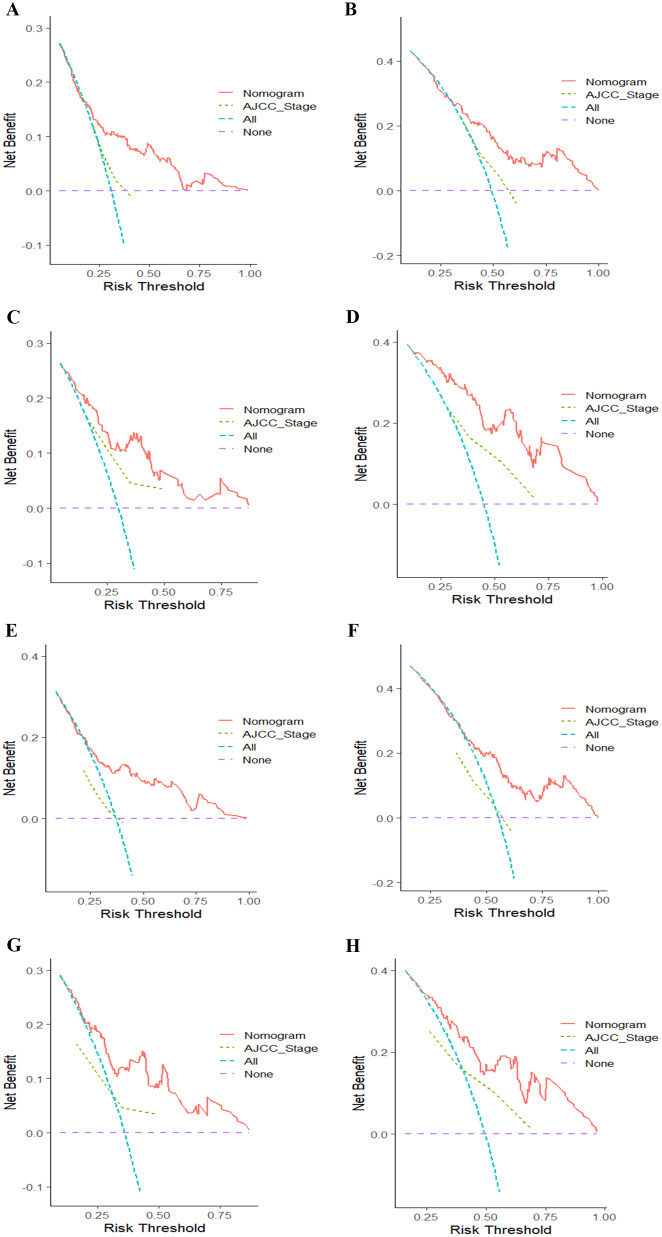
Decision curve analysis of the OS-associated and DFS-associated nomograms. DCA curves of 3-, and 5-year OS in the training cohort (**A**, **B**) and validation cohort (**C**, **D**). DCA curves of 3-, and 5-year DFS in the training group (**E**, **F**) and validation group (**G**, **H**). *Abbreviations* AJCC, American Joint Committee on Cancer; DCA, decision curve analysis; DFS, disease-free survival; OS, overall survival

The distinguishing capability of the two nomograms was certified by the ROC and the AUC value (Fig. 4). The AUC for predicting OS at 3-, and 5-year were 0.726, and 0.719 in the training cohort (Fig. 4A), and 0.806, and 0.827 in the validation cohort (Fig. 4B), respectively. Moreover, the 3-, and 5-year AUC for the prediction of DFS were 0.727, 0.718 (Fig. 4C), and 0.788, 0.808 (Fig. 4D) in the training group and validation group. Therefore, we can conclude that the newly developed nomograms had great differentiating capacity. Finally, the prediction performance of the two nomograms was confirmed by the DCA, which showed good positive net benefits and superior accuracy in comparison with the AJCC stage (Fig. 5).

### Comparison of clinical value between nomograms and AJCC stage system

In our study, C-index, NRI, and IDI were adopted to estimate the clinical application value of the two nomograms. Firstly, The NRI for the 3-, and 5-year OS were 0.287(95% CI: 0.224–0.432), 0.239(95% CI: 0.193–0.425) in the training group, and 0.410 (95% CI: 0.154–0.586), 0.434(95% CI: 0.207–0.604) in the validation group, respectively. The NRI for the 3-, and 5-year DFS in the training and validation sets were 0.284(95% CI: 0.211–0.428), 0.196(95% CI: 0.132–0.372), and 0.332(95% CI: 0.162–0.507), 0.427(95% CI: 0.166–0.564), respectively. Secondly, The IDI values for 3-, and 5-year OS were 0.151 (95% CI: 0.115–0.241, *P* < 0.001), 0.151 (95% CI: 0.103–0.221, *P* < 0.001) in the training cohort, and 0.137 (95% CI: 0.063–0.269, *P* < 0.001), 0.170 (95% CI: 0.076–0.289, *P* < 0.001) in the validation cohort. The IDI values for 3-, and 5-year DFS in the training group and validation group were 0.147 (95% CI: 0.106–0.220, *P* < 0.001), 0.121 (95% CI: 0.081–0.193, *P* < 0.001, and 0.123 (95% CI: 0.047–0.228, *P* < 0.001), 0.123 (95% CI: 0.054–0.236, *P* < 0.001), respectively. Finally, the C-index of the nomogram was also higher than that of the AJCC stage (0.691 vs. 0.580 in the training set, 0.722 vs. 0.554 in the validation set for predicting OS; 0.674 vs. 0.591, 0.680 vs. 0.553 in the training and validation groups for predicting DFS) (Table 4). In summary, all these results demonstrated that these two nomograms performed well compared with the AJCC stage.

### Risk stratification for postoperative patients with OCSCC

**Fig. 6:**
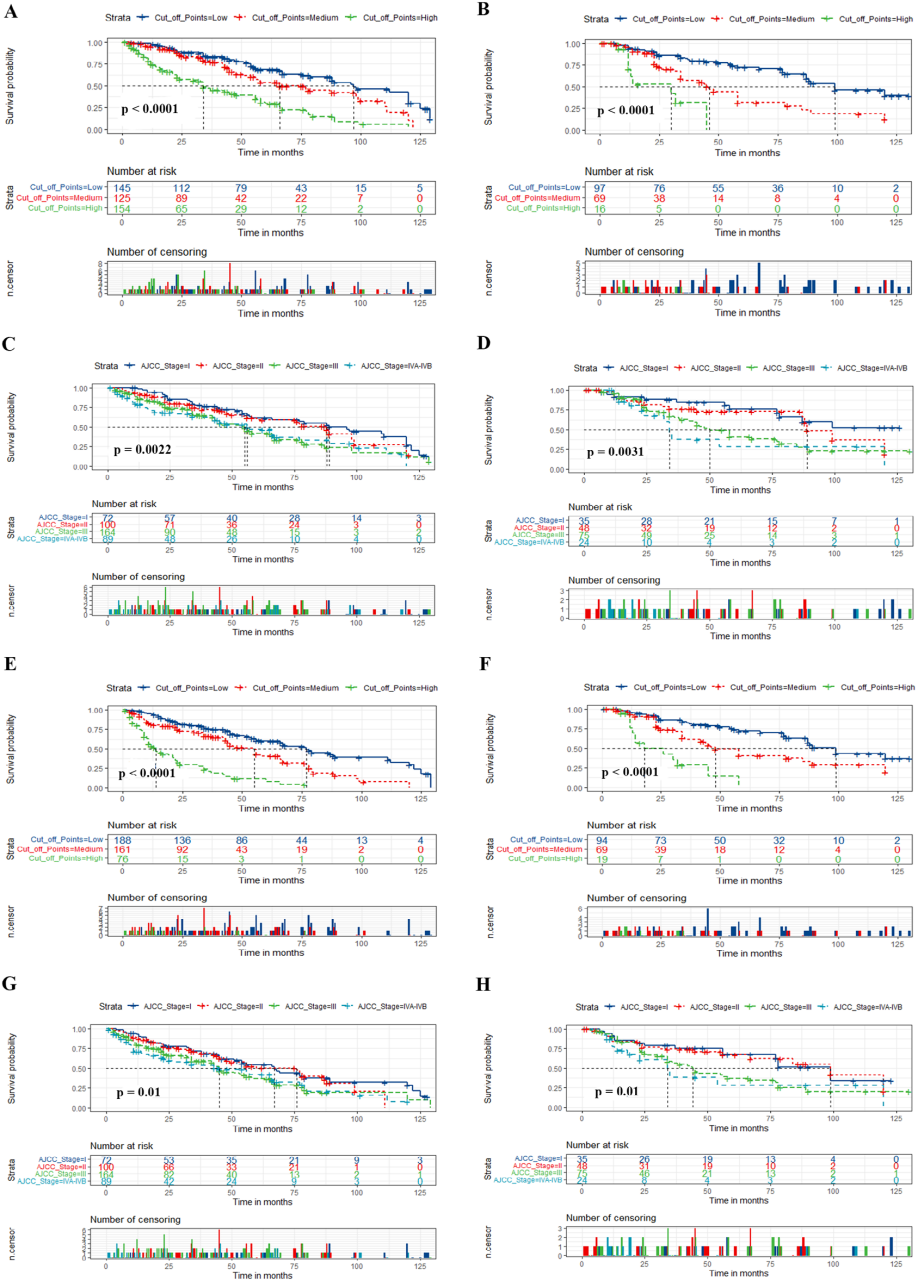
Kaplan-Meier curves of postoperative patients with OCSCC for predicting OS and DFS based on the new risk stratification system and the AJCC stage system. (**A**, **B**) Kaplan-Meier curves in the training (**A**) and validation cohorts (**B**) according to the new risk stratification system. (**C**, **D**) Kaplan-Meier curves according to the AJCC stage system of the training (**C**) and validation cohorts (**D**). (**E**, **F**) Kaplan-Meier DFS curves based on the new risk stratification system in the training (**E**) and validation cohorts (**F**). (**G**, **H**) Kaplan-Meier DFS curves according to the AJCC stage system in the training (**G**) and validation cohorts (**H**). *Abbreviations* AJCC, American Joint Committee on Cancer; DFS, disease-free survival; OS, overall survival; OCSCC, oral cavity squamous cell carcinoma

The risk stratification was divided according to the total score, which was calculated based on the two nomograms. All postoperative OCSCC patients were then classified into three risk cohorts for predicting OS and DFS (predicting OS: low-risk (points ≤ 165.31), medium-risk (165.32 ≤ points ≤ 237.45), and high-risk (points ≥ 507.37); for prediction of DFS: low-risk (points ≤ 157.46), medium-risk (157.47 ≤ points ≤ 241.66), and high-risk (points ≥ 482.00). There was significantly superior discrimination of OS and DFS in these three risk subgroups for both training and validation groups displayed by Kaplan-Meier curves in comparison to the traditional AJCC stage (Fig. 6). Figure 6C **&** Fig. 6D **and** Fig. 6G **&** Fig. 6H show the deficient capability of the AJCC stage system in differentiating mortality risk, notably for AJCC stage III and stage IVA & IVB, equally for AJCC stage I and stage II.

## Discussion

OCSCC is one of the most common types of oral cancer worldwide. The prevalence of OCSCC is high in low- and middle-income countries, especially in Asia populations who have special diets and lifestyle habits. Recently, the incidence and mortality of OCSCC have kept increasing, which has caused a profound effect on human health and life quality. The major treatment for OCSCC patients is a combination of surgery, radiotherapy, and chemotherapy. There were important differences between postoperative patients and unresectable patients with OCSCC in the treatment, prognosis, life quality, etc. Recently, a significant number of researchers focused their studies on the prognostic prediction of patients with OCSCC [22, 37, 38]. However, there was no prognostic model particularly for postoperative patients with OCSCC so far. It was a major issue for surgeons to optimize the clinical management in postoperative patients with OCSCC. Therefore, it is essential for us to pay more attention to this problem and establish a specialized prognostic model for postoperative patients with OCSCC in China populations.

In this study, a total of 9 independent prognostic variables were found to significantly correlated with OS and DFS in postoperative OCSCC patients by univariate and multivariate Cox regression analysis, including AJCC stage, perineural invasion, smoking after surgery, ECOG PS score, SII, PLR, ACCI, adjuvant radiotherapy, and adjuvant chemotherapy, which was largely consistent with previous analyses [25, 30, 39–42]. Then the two postoperative nomograms were established based on these discovered independent prognostic factors. Previously, researchers paid more attention to the prognosis of all OCSCC patients. A multi-institution researchers developed a new risk stratification for oral cancer patients in Southeast Asia [40]. Hai-Xuan Wu et al. [43] established a nomogram including inflammatory markers for prognosis prediction of primary OCSCC patients. Additionally, there are many differences between resectable OCSCC and unresectable OCSCC. A model for predicting prognosis in those patients is needed. Previously, Yang Liu et al. [44] established and validated a model for postoperative oral cavity carcinoma based on the SEER database. However, there were some limitations in this model as the patients are mainly from Europe and America, which might not be suitable for Chinese patients. The nomogram developed in this study could fulfill this requirement.

ECOG PS score was an important system to evaluate the overall behavior and activity of daily living in cancer patients, and there were few studies focused on the correlation between ECOG PS score and OCSCC. Yamada Shin-Ichi et al. [25] found that ECOG PS 2 or greater indicates a poor prognosis in elderly patients with primary OCSCC (75 years of age or older). ECOG PS score was not only important in elderly patients but also in other ages of patients with OCSCC. In this study, the ECOG PS score was found to be positively correlated with the prognosis of postoperative patients with OCSCC. Obviously, the higher the ECOG PS score, the worse the OS and DFS in patients with OCSCC.

Various studies demonstrated that perineural invasion was associated with poor DFS in OCSCC [45, 46]. In our study, perineural invasion was an important prognosis predictor of OS and DFS in postoperative OCSCC patients, which is consistent with the previous conclusions.

The ACCI is a comprehensive evaluation index of comorbidity and age, which has been reported to predict prognosis in various cancers [35, 36, 47]. Until now, the correlation between ACCI and OCSCC has not been studied. In this study, ACCI was first been incorporated into the analysis, which displayed significant importance in survival time prediction of OS and DFS for postoperative patients with OCSCC.

SII and PLR are immune-inflammation correlated clinicopathological indices, which were considered crucial predictors in OCSCC patients. Recently, Kosei Kubota et al. [31] found that higher SII was correlated with poorer DFS in patients with oral cancer. High PLR and SII indicate a worse progression-free survival, and disease-specific survival in OCSCC patients [48]. Similarly, our study found that PLR and SII were significant independent predictors in postoperative patients with OCSCC. For OS and DFS, high NLR and high SII were significantly associated with a worse prognosis, which is consistent with the results of previous studies.

Two nomograms for predicting OS and DFS were developed and validated in postoperative patients with OCSCC according to the data from two China tertiary medical centers. The validation results show that these two nomograms perform well and have excellent predictive and discriminative capability. As a result, two new risk stratification systems for postoperative patients with OCSCC were constructed, which displayed a good capability to distinguish risk groups in comparison with the traditional AJCC stage.

There are still some limitations indisputably in this research. Firstly, some important variables were not included in this study which resulted in some limitations, such as alcohol intake [41], P53 [49], and epidermal growth factor receptor (EGFR) [50]. Secondly, this study is a retrospective study, which has inevitable selection bias. Finally, data from other regions should be collected to improve persuasiveness, and prospective studies should be conducted to remedy these shortcomings in the future.

## Conclusion

Two nomograms and risk stratification systems were developed based on the cases from the central region of China, which display superior predictive efficacy and good net benefit in comparison with the AJCC stage. These new models can benefit clinicians and clinical practice.

### Electronic supplementary material

Below is the link to the electronic supplementary material.

Supplementary Material 1

Supplementary Material 2

## Data Availability

Detailed data are available from the corresponding author on reasonable request.

## Abbreviations

ACCI: Age-adjusted Charlson comorbidity index
AJCC: American joint committee on cancer
AUC: Area under curve
BMI: Body mass index
C-index: Consistency-index
DCA: Decision curve analysis
DFS: Disease-free survival
ECOG: Eastern cooperative oncology group
EGFR: Epidermal growth factor receptor
ENE: Extranodal extension
GPS: Glasgow prognostic score
HGB: Hemoglobin
HPV: Human papillomavirus
IDI: Integrated discrimination improvement
IQR: Interquartile range
LNM: Lymph node metastasis
NLR: Neutrophil-to-lymphocyte ratio
NRI: Net reclassification improvement
OS: Overall survival
OCSCC: Oral cavity squamous cell carcinoma
PLR: Platelet-to-lymphocyte ratio
PNI: Prognostic nutrition index
PS: Performance status
ROC: Receiver operating characteristic
RT: Radiotherapy
SD: Standard deviation
SII: Systemic immune-inflammation index
VI: Vascular invasion
