# Supplementary material for: Novel prognostic nomograms for postoperative patients with oral cavity squamous cell carcinoma in the central region of China
Source: BMC Cancer. 2024 Jun 14;24:730. doi: 10.1186/s12885-024-12465-6 (PMC11177417; doi:10.1186/s12885-024-12465-6)
Supplement: Supplementary file 1 — Supplementary Material 1 [file 12885_2024_12465_MOESM1_ESM.docx]

| **Table S1**. Age-adjusted Charlson Comorbidity Index. | |
| --- | --- |
| **Clinical conditions included in the score** | **Scores** |
|  | **Scores for disease** |
| Myocardial infarction; Peripheral vascular disease; Congestive heart failure; Dementia; Chronic pulmonary disease; Connective tissue disease; Diabetes; Cerebrovascular disease; Ulcer disease; Mild liver disease | 1 points |
| Hemiplegia; Diabetes with endo organ damage; Moderate or severe renal disease; Any tumor; Leukemia; Lymphoma | 2 points |
| Moderate or severe liver disease | 3 points |
| Metastatic solid tumor; Acquired immune deficiency syndrome | 6 points |
|  | **Scores for age** |
| Each decade over age 40 years (up to 4 points) | 1 points |
