# Supplementary material for: Novel prognostic nomograms for postoperative patients with oral cavity squamous cell carcinoma in the central region of China
Source: BMC Cancer. 2024 Jun 14;24:730. doi: 10.1186/s12885-024-12465-6 (PMC11177417; doi:10.1186/s12885-024-12465-6)
Supplement: Supplementary file 2 — Supplementary Material 2 [file 12885_2024_12465_MOESM2_ESM.docx]

| **Table S2**. Calculation formulas in this study. | |
| --- | --- |
| **Clinical index** | **Calculation formula** |
| SII | Platelet count × Neutrophil count / lymphocyte count |
| GPS | Score of 0 if C-reactive protein ≤ 10 mg/dL and albumin ≥ 35 g/L  Score of 1 if C-reactive protein > 10 mg/dL or albumin < 35 g/L  Score of 2 if C-reactive protein > 10 mg/dL and albumin < 35 g/L |
| PNI | Serum albumin (g/L) + (lymphocyte count ×5) |
| NLR | Neutrophil count / lymphocyte count |
| PLR | Platelet count / lymphocyte count |
| BMI | Mass (kg) / height^2^(m^2^) |
| Abbreviation: BMI, body mass index; GPS, Glasgow prognostic score; PLR, platelet-to-lymphocyte ratio; PNI, prognostic nutrition index; NLR, neutrophil-to-lymphocyte ratio; SII; systemic immune-inflammation index. | |
